# Effectiveness of Hyperbaric Oxygen Therapy for Musculoskeletal Pain Syndromes: A Systematic Review

**DOI:** 10.3390/muscles4040063

**Published:** 2025-12-16

**Authors:** Sebastián Eustaquio Martín Pérez, Eduardo Iboleón Laynez, José Antonio Acevedo Rodríguez, María Isabel Maggioni Torres, Roberto Pérez Betancort, Antón Díaz Rodríguez, Diego Cabezos Alonso, Carlos García Camacho, Isidro Miguel Martín Pérez

**Affiliations:** 1 Faculty of Health Sciences, Universidad Europea de Canarias, 38300 La Orotava, Santa Cruz de Tenerife, Spain; adiazro11@gmail.com (A.D.R.);; 2 Escuela de Doctorado y Estudios de Posgrado, Universidad de La Laguna, 38203 San Cristóbal de La Laguna, Santa Cruz de Tenerife, Spain; 3 Faculty of Medicine, Health and Sports, Universidad Europea de Madrid, Villaviciosa de Odón, 28670 Madrid, Spain; 4 Club Baloncesto 1939 Canarias SAD, 38205 San Cristóbal de La Laguna, Santa Cruz de Tenerife, Spain; 5 Club Deportivo Tenerife Femenino SAD, 38002 Santa Cruz de Tenerife, Santa Cruz de Tenerife, Spain; 6 Powerguanche Strength Lab, 38004 Santa Cruz de Tenerife, Santa Cruz de Tenerife, Spain

**Keywords:** hyperbaric oxygen therapy, muscles, pain, fatigue, sports, rehabilitation

## Abstract

Background: Musculoskeletal pain syndromes (MPSs) represent a major cause of disability and reduced quality of life, and conventional therapeutic approaches often provide only partial or temporary relief. Hyperbaric oxygen therapy (HBOT) delivered as 100% oxygen at 1.3–2.5 ATA, has been proposed to modulate inflammatory processes and enhance tissue repair. This review evaluated the effectiveness of HBOT on pain, function, quality of life, and physiological outcomes in individuals with MPS. Methods: This systematic review was conducted in different databases between June 30 and 30 September 2025, following PRISMA guidelines and was previously registered in PROSPERO (CRD420251073730). Studies published in English, Spanish, or Portuguese evaluating HBOT as a standalone or adjunctive intervention were included. Methodological quality and risk of bias were assessed using PEDro, NIH, and RoB 2.0 tools, and certainty of evidence was graded with GRADE. Results: Eighteen studies (17 RCTs and 1 case series; *n* = 671) were included. HBOT protocols ranged from 3 to 60 sessions, lasting 60–90 min, at approximately 1.3–2.5 ATA. Consistent reductions in pain and modest functional improvements were observed in fibromyalgia and postoperative conditions such as knee arthroplasty and peripheral nerve repair, with associated improvements in quality of life and inflammatory markers. Results for delayed-onset muscle soreness and acute ligament injuries were inconsistent. Conclusions: HBOT may provide adjunctive benefits in musculoskeletal pain syndromes, yet the current evidence remains limited. Standardized treatment protocols and high-quality trials are needed to better define its clinical applicability.

## 1. Introduction

Musculoskeletal pain syndromes are globally widespread, affecting an estimated 20–33% of the adult population, and contributing to more than 1.7 billion cases of musculoskeletal-related disability worldwide [1,2]. Mechanistically, MPSs involve a combination of peripheral tissue alterations, such as microvascular dysfunction, localized hypoxia, and low-grade inflammation, together with central sensitization and impaired descending pain modulation [3,4]. These processes sustain pain and lead to stiffness, fatigue, and reduced functional capacity, which negatively affect daily activities, exercise tolerance, and occupational performance. Importantly, MPSs encompass both chronic nociplastic conditions, such as Fibromyalgia syndrome, and acute or subacute manifestations, including delayed-onset muscle soreness (DOMS), exercise-induced muscle damage, and sports-related neuromuscular injuries [5,6,7].

From a therapeutic perspective, conservative management can be divided into pharmacological and non-pharmacological approaches. Pharmacological treatment is typically used for pain control, while non-pharmacological interventions include therapeutic exercise [8], manual therapy [9], cryotherapy [10], and electrostimulation [11], all aimed at pain relief and functional recovery. However, the clinical efficacy of these approaches is often inconsistent, particularly in recurrent or chronic cases, and they offer limited capacity to directly modulate the hypoxic and inflammatory milieu that underlies many MPS presentations [12,13].

In this therapeutic context, hyperbaric oxygen therapy (HBOT) has been proposed as a promising adjunct treatment [14,15,16]. HBOT consists of breathing 100% oxygen within a pressurized chamber (typically ranging from 1.3 to 3.0 atmospheres absolute, ATA), which significantly elevates oxygen partial pressure in arterial blood and enhances oxygen diffusion into peripheral tissues [17,18]. In both acute and chronic presentations, MPSs are often associated with transient microvascular ischemia, oxidative stress, localized inflammation, and reduced oxygen availability in the affected musculature. By enhancing tissue oxygenation, HBOT is thought to counteract hypoxia-related stress and inflammatory dysregulation, improve microcirculatory function, and support cellular repair processes [14,15,19]. Consequently, it may mitigate conditions such as DOMS and facilitate post-exercise recovery through sustained support of oxygen-dependent muscle regeneration mechanisms [17,20,21].

Several clinical studies have explored the application of HBOT across a range of musculoskeletal conditions, including Fibromyalgia syndrome, ankle sprains, contusions, post-exercise fatigue, and muscle–tendon injuries [18,19,22,23,24]. Although these studies suggest potential therapeutic benefits, the evidence base remains fragmented and difficult to understand. Substantial heterogeneity persists in study design, sample characteristics, HBOT dosing parameters (e.g., pressure, session duration, and total treatment volume), and the timing and selection of outcome measures. This methodological variability limits cross-study comparability and constrains the ability to draw consistent, clinically meaningful conclusions.

Despite growing clinical and research interest in HBOT within rehabilitation and sports medicine, its effectiveness across the broader spectrum of MPS has not yet been synthesized in a comprehensive, systematic and integrative manner. The absence of such synthesis impedes the development of evidence-based recommendations and restricts the establishment of clinical pathways necessary for routine implementation. Therefore, the present review aims to critically appraise and synthesize the available evidence on the effects of HBOT in individuals with MPS, with a particular focus on outcomes related to pain reduction and functional recovery.

## 2. Results

### 2.1. Study Selection

**Figure 1:**
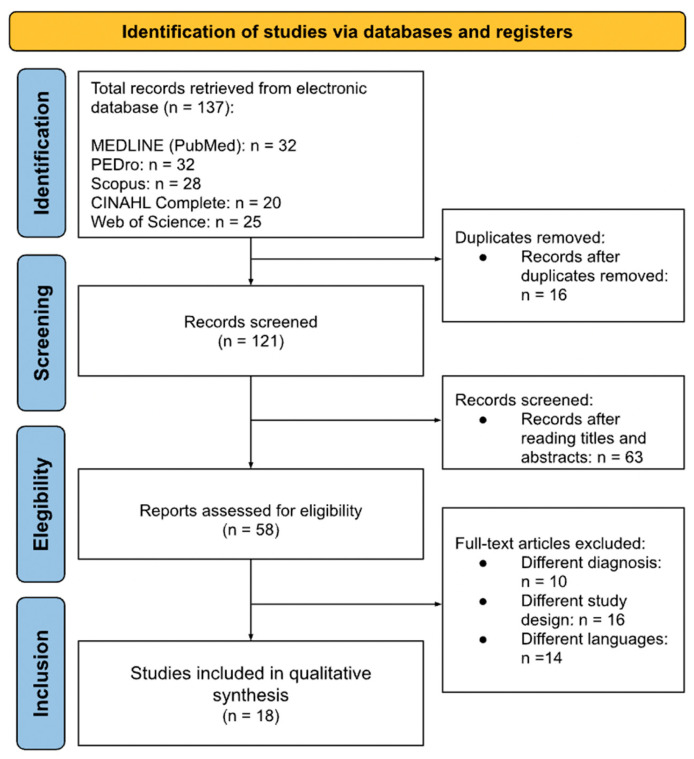
PRISMA 2020 flow diagram [25] of study selection. A total of 137 records were identified (MEDLINE (*PubMed*), PEDro, Scopus, CINAHL, Web of Science); 16 duplicates were removed. After screening 121 records and assessing 58 full texts, 18 studies met the inclusion criteria.

A total of 137 records were retrieved through database searches: MEDLINE (*PubMed*) (*n* = 32), PEDro (*n* = 32), Scopus (*n* = 28), CINAHL Complete (*n* = 20), and Web of Science (*n* = 25). After removing 16 duplicates, 121 records remained for title and abstract screening. During this screening, 63 records were excluded, and 58 full-text articles were assessed for eligibility. Of these, 40 articles were excluded due to non-eligible diagnoses (*n* = 10), inappropriate study designs (*n* = 16), or publication in non-eligible languages (*n* = 14). Ultimately, 18 studies met the inclusion criteria and were included in the qualitative synthesis. The selection process is depicted in Figure 1 as a PRISMA flow diagram.

### 2.2. Study Characteristics

A total of 18 studies [26,27,28,29,30,31,32,33,34,35,36,37,38,39,40,41,42,43,44] were included, comprising 17 randomized controlled trials and 1 retrospective case series, with a combined sample of *n* = 671 participants. The studies covered various MPS and injury conditions, including fibromyalgia [30,32,35,36], overuse and exercise-related pain [27,28,31,37,38,39,42], DOMS [37,38,40,41,42], post-TKA recovery [26], acute ankle and knee ligament injuries [33,43], and ulnar/median nerve injuries [29]. Both sexes were represented, although reporting was inconsistent.

Regarding study design, 7 trials used a single-blind approach [26,27,37,38,39,41,43], 6 were double-blind RCTs [28,30,31,35,36,42], and 2 used a crossover design [32,35]. One study was a retrospective case series [34], and the remainder followed standard parallel-group RCT designs. Further, HBOT protocols ranged from 1.3 to 2.5 ATA, with sessions lasting 60–90 min.

Treatment duration varied from 3 to 15 sessions [26,27,29,33,36,37,38,39,41,43] to 40–60 sessions in longer interventions [28,30,32,35]. Some studies combined HBOT with physical training, resistance exercise, or PRP injections [27,30,34]. Control conditions included sham sessions, normobaric oxygen, delayed intervention, or no additional treatment [26,27,28,29,30,31,32,33,34,35,36,37,38,39,40,41,43]. Outcome measures included biomarkers of muscle damage and inflammation (e.g., CK, LDH, myoglobin, CRP, IL-6, TNF-α) [26,27,34,37], oxidative stress markers (e.g., MDA, SOD, CAT, protein carbonyls, T-AOC) [27,37], and clinical pain outcomes such as VAS, tender point counts, and pressure pain threshold (PPT) [26,28,29,30,31,32,33,34,35,36,37,38,39,41,42,43,44].

Functional outcomes included measures of muscle performance (e.g., quadriceps and isokinetic strength, torque, MVC), physical function (e.g., 6 MWT, SPPB, functional indices), joint status (e.g., ROM, swelling, limb circumference), and time to pain-free walking [26,27,28,29,31,33,34,37,38,39,40,41,42,43,44]. Additional outcomes encompassed recovery time and recurrence [34], quality of life and psychological scales (e.g., FIQ, SF-36, WPI, SSS, CAPS, SCL-90) [30,32,35], and neuroimaging assessments (e.g., MRI, SPECT, DTI) [32,35,37,38,39].

The studies included in this review were conducted across a wide range of geographical regions, including China [26,27,31,33], Israel [28,32,35], Turkey [29,36], Spain [30], South Africa [34], Canada [37,38,39,44], the United States [40,43], Slovenia [41], and Australia [42]. Full study characteristics are presented in Table S2.

### 2.3. Methodological Quality Assessment: PEDro Scale

The methodological quality of the 18 randomized controlled trials, assessed with the PEDro scale [45], resulted in a mean score of 7.8 out of 10. As shown in Table 1 six studies were rated as excellent quality [28,30,31,36,41,42], four as moderate quality [26,32,39,43], seven as good quality [27,29,35,37,38,40,44], and one as low quality [33]. Across these studies [26,27,28,29,30,31,32,33,35,36,37,38,39,40,41,42,43,44], the main methodological strengths included clearly defined eligibility criteria, random allocation, comparability between groups at baseline, and the blinding of outcome assessors. Conversely, the most common limitations were the absence of blinding of participants and therapists, together with incomplete allocation concealment.

According to the NIH Quality Assessment Tool for Case Series Studies, the case series by Botha et al. [34] was rated as having low to moderate methodological quality, with strengths in the clarity of objectives, detailed population description, and intervention reporting, but notable limitations in consecutive case inclusion, comprehensive follow-up, and the absence of any blinding procedures. A detailed description of the scoring criteria and ratings is also provided in Table 2.

### 2.4. Risk of Bias Assessment: RoB 2.0

The overall risk of bias, assessed using the Cochrane Risk of Bias 2.0 tool [47], ranged from low to moderate. A moderate risk in the randomization process was observed in studies with insufficient reporting of allocation concealment [27,31,36,41,42]. Additionally, a moderate to high risk related to deviations from intended interventions was identified in studies that did not implement blinding of participants and personnel [29,31,33,36,40,43].

**Figure 2:**
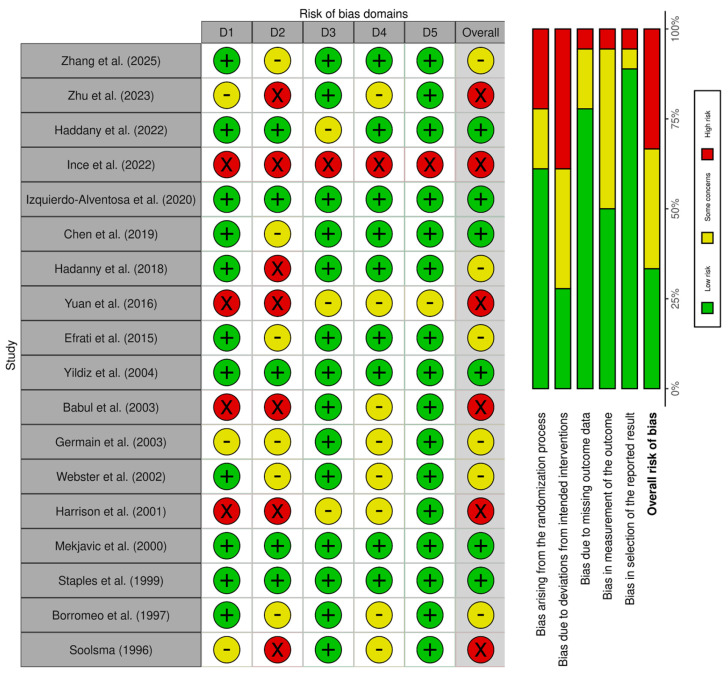
Risk of bias assessment of the included studies according to the Cochrane Risk of Bias tool (RoB 2.0) [47]. Color coding: Green = Low risk of bias; Yellow = Some concerns; Red = High risk of bias. Abbreviations: D1 = Bias arising from the randomization process; D2 = Bias due to deviations from intended interventions; D3 = Bias due to missing outcome data; D4 = Bias in the measurement of the outcome; D5 = Bias in the selection of the reported result; Overall = Overall risk of bias judgment. Studies included: Zhang et al. (2025) [26], Zhu et al. (2023) [27], Hadanny et al. (2022) [28], Ince et al. (2022) [29], Izquierdo-Alventosa et al. (2020) [30], Chen et al. (2019) [31], Hadanny et al. (2018) [32], Yuan et al. (2016) [33], Efrati et al. (2015) [35], Yildiz et al. (2004) [36], Babul et al. (2003) [37], Germain et al. (2003) [38], Webster et al. (2002) [39], Harrison et al. (2001) [40], Mekjavic et al. (2000) [41], Staples et al. (1999) [42], Borromeo et al. (1997) [43], and Soolsma (1996) [44].

With respect to missing outcome data, most studies demonstrated low risk, supported by adequate follow-up and appropriate handling of attrition [26,27,28,29,30,31,32,33,34,35,36,37,38,39,40,41,42,43,44]. A moderate risk in outcome measurement was noted in studies without blinded outcome assessment [29,31,33,36]. Importantly, all studies showed low risk for selective reporting, with predefined outcomes consistently reported [26,27,28,29,30,31,32,33,34,35,36,37,38,39,40,41,42,43,44]. Moreover, more recent studies tended to demonstrate greater methodological rigor, resulting in lower overall risk profiles [26,27,28,29,30]. A summary of the risk of bias assessment is presented in Figure 2.

### 2.5. Certainty of Evidence: GRADE

The certainty of evidence for the effects of HBOT in musculoskeletal pain and injury was evaluated using the GRADE framework [48]. Overall, the certainty was rated as low across all outcome domains, including pain, functional recovery, quality of life, physiological markers, and return to sport. A total of 13 studies involving *n* = 422 participants reported outcomes related to pain intensity, encompassing chronic pain in Fibromyalgia syndrome, postoperative pain, and exercise-induced muscle soreness. Functional recovery was assessed in 9 studies with *n* = 419 participants, evaluating domains such as range of motion, mobility, strength restoration, and sensorimotor function following surgical or musculoskeletal injury.

Outcomes related to quality of life were reported in 4 studies (*n* = 180), with measurement tools including the SF-36, fatigue scales, sleep quality indicators, and psychological distress assessments, particularly in populations with Fibromyalgia. In addition, physiological markers—including inflammatory biomarkers, nerve conduction parameters, tissue oxygenation, and brain activity imaging—were documented in 8 studies involving *n* = 241 participants. Finally, return to sport or physical activity was evaluated in 5 studies (*n* = 159), focusing on recovery timelines following muscle injuries, ankle sprains, or nerve repair procedures.

Across all domains, the certainty of evidence was consistently downgraded due to serious risk of bias (e.g., small sample sizes, lack of blinding) and serious inconsistency (heterogeneous protocols and outcome measures). No serious concerns were identified regarding indirectness, imprecision, or publication bias. Therefore, the overall strength of recommendation for HBOT in musculoskeletal conditions remains weak, suggesting its use as an adjunctive rather than standalone intervention. Complete GRADE assessments for each outcome are presented in Table 3.

### 2.6. Data Synthesis

#### 2.6.1. Efficacy of HBOT vs. Control Interventions for *Pain Reduction* in Musculoskeletal Painful Syndromes

HBOT has been evaluated as an adjunctive intervention for MPS in both acute and chronic conditions. Across the available evidence, the most robust and consistent analgesic effects are observed in Fibromyalgia syndrome, whereas findings for exercise-induced pain, acute ligamentous injuries, and postoperative pain remain more heterogeneous and less definitive.

In Fibromyalgia, five clinical trials consistently documented improvements in pain-related outcomes. HBOT administered at 1.45 ATA for 90 min per session, 5 days per week, over 40 sessions was associated with lower VAS pain scores compared with controls (4.88 ± 2.32 vs. 5.50 ± 2.25; *n* = 17 and *n* = 16, respectively) [30]. Protocols involving approximately 2.0 ATA for 90 min per session with air breaks over 60 sessions achieved marked reductions in the Widespread Pain Index (15.27 ± 1.6 to 7.53 ± 3.7) and Symptom Severity Scale (11.5 ± 1.8 to 5.13 ± 2.8) [32].

Treatment at 2.0 ATA for 40 sessions similarly reduced the number of tender points (17.3 to 8.9) and increased pressure pain thresholds (0.55 to 1.65 kg/cm^2^) without comparable improvements in controls (*n* = 24) [35]. Moreover, a 2.4 ATA protocol for 15 sessions significantly reduced tender point counts (6.04 ± 1.18 vs. 12.54 ± 1.10) and VAS pain scores (31.54 ± 8.34 vs. 55.42 ± 6.58) compared with placebo (*n* = 24) [36]. In athletes with chronic musculoskeletal pain, HBOT at 2.5 ATA resulted in lower VAS scores (1.4 ± 1.7 vs. 3.3 ± 1.8) [31]. Taken together, these findings indicate a consistent and clinically meaningful analgesic effect of HBOT in chronic centralized pain conditions.

In contrast, evidence regarding pain associated with overuse or eccentric exercise was inconsistent. Studies applying 2.5 ATA demonstrated reductions in pain scores (1.4 ± 1.7 vs. 3.3 ± 1.8; *n* = 41) [31] and improvements in gastrocnemius strain pain (3.1 ± 1.1 vs. 4.8 ± 1.2; *p* < 0.05) after three sessions [39]. However, multiple trials employing 2.0–2.5 ATA over 4–10 sessions in the context of DOMS reported no significant between-group differences [37,38,40,41,42], suggesting that HBOT does not confer consistent benefit in exercise-induced muscle pain and may have limited value in conditions characterized by transient inflammatory overload.

Findings in postoperative and trauma-related pain suggest potential benefits, although results remain variable. HBOT at 2.0 ATA across 5 postoperative sessions led to lower pain scores on postoperative days 2–3 compared with normobaric oxygen (*n* = 40 per group) [26]. Likewise, HBOT at 2.0 ATA for 2 h per day over 5 days following median and ulnar nerve repair improved sensory recovery and increased nerve conduction velocities relative to controls [29].

By contrast, studies addressing acute ligamentous injuries reported limited analgesic benefit. HBOT at 2.0 ATA for three sessions within 72 h after ankle sprain resulted in non-significant VAS differences (1.7 ± 1.7 vs. 2.1 ± 1.5) [43], and treatment at 2.5 ATA for 10 sessions in grade II medial collateral ligament injury yielded similarly comparable pain outcomes (1.71 ± 1.38 vs. 1.86 ± 1.46) [44]. Taken together, these findings indicate that HBOT does not appear to provide a clinically meaningful advantage in the management of acute ligamentous injuries.

#### 2.6.2. Effectiveness of HBOT Versus Control on *Functional Recovery* in Musculoskeletal Painful Syndromes

HBOT has also been investigated for its effects on functional recovery and physical performance, in addition to pain reduction in MPS. In Fibromyalgia, improvements in functional status appear to accompany reductions in pain intensity. For example, in a protocol of ~2.0 ATA for 90 min with periodic air breaks across 60 sessions, functional capacity (FIQ) improved markedly, decreasing from 69.6 ± 9.2 at baseline to 36.5 ± 13.4 post-treatment, indicating enhanced daily functioning and reduced disability [32]. Similarly, a 2.0 ATA protocol delivered over 40 sessions was associated not only with reductions in tender points and increased pressure pain thresholds, but also with improvements in cognitive performance and quality-of-life scores [35]. These findings suggest that HBOT may exert broader effects on function by modulating both pain perception and neurocognitive processing in Fibromyalgia.

In exercise-related conditions, functional recovery outcomes were more variable but showed some favorable effects. In athletes treated at 2.5 ATA (100% oxygen), return to pre-exercise strength and flexibility occurred in 3.2 ± 0.9 days, compared with 5.8 ± 1.1 days in controls [31]. Similarly, athletes with gastrocnemius strain receiving three sessions at 2.5 ATA were able to resume training approximately two days earlier than those in the control group [39]. These results indicate that HBOT may facilitate recovery in high-demand musculoskeletal scenarios, although the evidence base remains limited to small sample studies.

More consistent benefits in functional recovery have been reported in postoperative and nerve injury rehabilitation. Following total knee arthroplasty, HBOT at 2.0 ATA for five postoperative sessions improved knee range of motion by day 7 (115.4 ± 8.2° vs. 108.1 ± 7.9°) relative to normobaric oxygen [26]. Likewise, in patients undergoing median and ulnar nerve repair, HBOT at 2.0 ATA for 2 h/day over five sessions initiated immediately after surgery enhanced motor conduction velocities and accelerated sensory recovery compared with standard care [29]. These findings suggest that HBOT may support functional restoration, particularly where tissue healing, neural recovery, and inflammatory modulation are central.

In contrast, trials evaluating acute ligamentous injuries reported no significant differences in time to return to sports or functional scores between HBOT and control groups [43,44]. Overall, the current evidence indicates that HBOT provides the most consistent functional recovery benefit in Fibromyalgia and post-surgical rehabilitation, with more limited and inconclusive effects in exercise-induced muscle injury and acute ligamentous trauma.

#### 2.6.3. Effectiveness of HBOT Versus Control on *Quality of Life* in Musculoskeletal Painful Syndromes

HBOT has also been evaluated for its effects on health-related quality of life (HRQoL) and psychological well-being, dimensions that commonly accompany MPS. Overall, the available evidence indicates that HBOT confers clinically relevant improvements in HRQoL and psychological status in chronic musculoskeletal pain conditions, particularly in Fibromyalgia syndrome, whereas findings in acute musculoskeletal injuries remain limited and inconclusive.

In Fibromyalgia, several trials have reported substantial enhancements in HRQoL following HBOT. For instance, treatment protocols involving approximately 2.0 ATA for 90 min per session across 60 sessions led to a marked reduction in FIQ scores, decreasing from 69.6 ± 9.2 at baseline to 36.5 ± 13.4 post-intervention, with improvements exceeding 40% in domains related to fatigue, morning tiredness, and depressive symptoms [32]. Similarly, 40 sessions at 2.0 ATA resulted in significant increases in SF-36 vitality (38 ± 12 to 62 ± 14) and mental health (40 ± 11 to 65 ± 13) subscale scores, alongside measurable gains in cognitive performance [35].

Sleep-related outcomes also improved, as demonstrated by a 3.2-point reduction in Pittsburgh Sleep Quality Index scores compared with controls [30]. In athletes with chronic pain, HBOT at 2.5 ATA was associated with reductions in perceived fatigue and improved mood status, with the tension-anxiety subscale of the Profile of Mood States decreasing from 13.2 ± 5.1 to 6.5 ± 3.9 [31]. Taken together, these findings indicate that HBOT may positively influence psychological well-being through mechanisms linked to pain modulation, sleep regulation, and cognitive-emotional processing.

In postoperative and peripheral nerve injury contexts, improvements in HRQoL have also been reported, although outcome measures were less standardized. Following total knee arthroplasty, enhanced pain control with HBOT was accompanied by higher early recovery satisfaction and improved emotional well-being [26]. Similarly, patients undergoing median and ulnar nerve repair reported subjective improvements in sleep and mood during postoperative recovery when treated with HBOT at 2.0 ATA [29]. By contrast, studies assessing acute ligamentous injuries did not demonstrate significant between-group differences in psychological well-being or HRQoL measures [43,44].

Overall, the evidence indicates that HBOT exerts the most consistent HRQoL benefits in chronic centralized pain conditions, particularly Fibromyalgia, whereas effects in acute ligamentous injury and short-term trauma contexts remain inconclusive.

#### 2.6.4. Effectiveness of HBOT Versus Control on *Physiological Markers* in Musculoskeletal Painful Syndromes

In addition to clinical outcomes, several studies have examined the effects of HBOT on physiological and biological markers relevant to MPS. In Fibromyalgia, 40 sessions of HBOT at 2.0 ATA (100% oxygen, 90 min per session, five sessions per week) were associated with significant neuroplastic changes. Post-treatment SPECT and diffusion tensor imaging showed increased activity in frontal, subcortical, and thalamic regions, as well as improved white matter integrity, changes that correlated with reductions in tender point count, increased pressure pain thresholds, and functional improvements [35]. These findings suggest that HBOT may exert therapeutic effects in Fibromyalgia at least in part through modulation of central nociceptive processing.

In peripheral nerve injury, HBOT at 2.0 ATA for 2 h per day over five consecutive days beginning on the first postoperative day following epineural repair resulted in measurable improvements in neural conduction. At three months, patients treated with HBOT exhibited significantly higher median nerve motor conduction velocities (53.2 ± 5.8 m/s vs. 48.5 ± 6.1 m/s; *p* < 0.01) and larger sensory nerve action potentials (12.4 ± 3.1 µV vs. 8.9 ± 2.8 µV) compared with controls, indicating accelerated and more complete neural recovery [29].

In the postoperative setting, HBOT at 2.0 ATA (100% oxygen, five sessions initiated immediately after total knee arthroplasty) was associated with reduced thigh swelling and lower levels of inflammatory biomarkers, including CRP, IL-6, and TNF-α, during the first postoperative week [26]. These reductions are consistent with enhanced tissue oxygenation, modulation of inflammatory cascades, and improved recovery trajectories.

In exercise-related muscle injury, three sessions of HBOT at 2.5 ATA resulted in reduced muscle edema on ultrasound imaging following gastrocnemius strain [39]. However, other studies investigating DOMS using 2.0–2.5 ATA protocols did not include mechanistic biomarker assessments and did not show physiological effects beyond subjective outcomes, limiting interpretations in this domain [37,38,40,41,42].

Overall, the evidence indicates that HBOT may enhance neural conduction, modulate central neuroplasticity, and attenuate postoperative inflammatory responses, particularly in Fibromyalgia and peripheral nerve repair. By contrast, mechanistic data in acute ligamentous injury and exercise-induced muscle damage remain scarce and inconclusive, highlighting the need for targeted physiological and imaging endpoints in future trials.

#### 2.6.5. Effectiveness of HBOT Versus Control on *Return-to-Sport Time* and *Performance Recovery* in Musculoskeletal Painful Diseases

HBOT has also been evaluated for its effects on return-to-sport timelines and recovery of physical performance, particularly among athletes and individuals with MPS. In exercise-induced and overuse conditions, HBOT at 2.5 ATA (100% oxygen) was associated with accelerated recovery: athletes regained pre-exercise strength and flexibility in 3.2 ± 0.9 days compared with 5.8 ± 1.1 days in controls [31]. Likewise, in gastrocnemius strain, three sessions of HBOT at 2.5 ATA enabled an earlier return to normal training by approximately two days relative to control treatment [39]. These findings suggest that HBOT may facilitate recovery in high-demand physical performance contexts where rapid return to baseline function is a priority.

However, evidence from DOMS trials was consistently negative. Studies applying 2.0–2.5 ATA across 4–10 sessions did not detect significant differences in recovery timelines, restoration of muscle strength, or return to baseline performance when comparing HBOT with control conditions [37,38,40,41,42]. In these contexts, the magnitude and duration of muscle microtrauma may be insufficient to benefit meaningfully from HBOT-induced physiological changes, contributing to the lack of demonstrated effect.

In postoperative and peripheral nerve repair settings, HBOT appears to support earlier functional recovery milestones. Following total knee arthroplasty, five postoperative sessions at 2.0 ATA resulted in earlier achievement of independent standing and walking, occurring by day 5 in the HBOT group compared with day 7 in controls [26]. Similarly, HBOT at 2.0 ATA for 2 h/day across five sessions initiated immediately after median and ulnar nerve repair was associated with earlier recovery of functional hand use, consistent with improvements in nerve conduction and sensory recovery, although exact timelines were not specified [29]. These findings align with the physiological evidence of improved tissue oxygenation, reduced inflammation, and enhanced neural regeneration associated with HBOT.

By contrast, in acute ligamentous injuries, no significant differences have been observed in return-to-sport or performance recovery outcomes. Studies administering HBOT either at 2.0 ATA for three sessions after ankle sprain [43] or at 2.5 ATA for ten sessions in grade II medial collateral ligament injury [44] reported similar recovery durations in HBOT and control groups, indicating a lack of clinically relevant benefit in these injury types. Overall, HBOT demonstrates the most consistent benefit for accelerating return-to-function in exercise-related overuse injuries and postoperative rehabilitation, while effects on DOMS and acute ligamentous injuries remain inconclusive. A summary of these findings is provided in Table 4.

## 3. Discussion

This systematic review examined the potential role of HBOT as an adjunctive therapy in chronic musculoskeletal pain conditions. Overall, the certainty of the evidence remains low to moderate, largely due to small sample sizes, heterogeneity in treatment protocols, and variability in outcome measures. Despite these limitations, several studies reported clinically meaningful improvements, suggesting that HBOT may confer therapeutic benefit in selected patient populations. Nonetheless, these findings should be interpreted with caution.

Previous systematic reviews addressing HBOT for musculoskeletal pain have primarily focused on Fibromyalgia Syndrome [49,50] or postoperative rehabilitation [18]. These works have generally been limited to summarizing clinical outcomes, without considering the underlying pathophysiological mechanisms that characterize chronic pain states. In contrast, the present review adopts a broader analytical framework, encompassing both chronic and acute musculoskeletal conditions and integrating clinical outcomes with current knowledge on neurobiological sensitization, inflammatory modulations, and tissue repair pathways. This approach provides a more comprehensive interpretive context for understanding how HBOT may influence pain and functional recovery.

However, consistent with previous reviews, the overall strength of the evidence remains limited. This reinforces the need for larger, methodologically rigorous randomized controlled trials, employing standardized HBOT dosages, comparable outcome metrics, and long-term follow-up to better establish both efficacy and durability of treatment effects. As noted above, previous trials investigating HBOT have primarily focused on Fibromyalgia Syndrome, a condition frequently associated with central sensitization [51]. These studies consistently reported reductions in pain intensity and tender point sensitivity, reflected in improvements in VAS scores, WPI values, and tender point indices [30,32,35,36].

While these outcomes appear clinically meaningful, the explanatory strength of this hypothesis remains limited due to methodological heterogeneity and variation in follow-up periods across studies. Nevertheless, the overall pattern of findings supports the proposition that HBOT may modulate central pain processing mechanisms, contributing to reductions in symptom burden. Moreover, these results are conceptually coherent with the broader hypothesis that HBOT influences pain modulation networks implicated in central sensitization [52,53].

Likewise, neuroimaging studies in patients undergoing HBOT have reported alterations in metabolic activity within frontal cortical and thalamic regions, together with indications of changes in white matter microstructure [32,35,54,55,56,57,58,59,60,61]. Such patterns have been interpreted as potential markers of neuroplastic adaptation. Nevertheless, the evidence remains constrained by the limited number of studies available and their modest sample sizes, and therefore any mechanistic interpretations should be regarded as provisional. Similarly, preliminary molecular data suggesting modulation of neurotrophic signaling pathways (e.g., BDNF) and neuroinflammatory processes must be considered exploratory until confirmed by larger, adequately powered clinical trials [57,62].

Beyond its analgesic effects, HBOT has also been associated with functional improvements during post-operative rehabilitation and in chronic musculoskeletal conditions, including recovery following total knee arthroplasty (TKA) [26] and injuries arising from repetitive mechanical overload [27,28,31,37,38,39,42,63]. Proposed biological mechanisms indicate that increased tissue oxygen availability may facilitate angiogenesis, enhance fibroblast activity, and promote extracellular matrix repair and collagen remodeling [64,65,66]. Moreover, findings from neuropathic pain models—such as improvements in nerve conduction and reductions in pro-inflammatory cytokines (e.g., IL-6, TNF-α, CRP)—lend additional biological plausibility, although the certainty of this evidence remains limited [26,29,54,55,67].

Importantly, quality-of-life improvements in Fibromyalgia—such as reductions in FIQ scores and improvements in sleep, fatigue, and mood [30,32,35]—also warrant careful interpretation. Proposed mechanisms involving enhanced cerebral oxygenation, mitochondrial bioenergetics, and neurochemical stabilization in affective pain-regulation regions remain hypothetical and lack support from large, adequately powered trials. The observed normalization of oxidative and inflammatory biomarkers may contribute to reduced psychological distress and central sensitization symptoms, although these findings, too, reflect preliminary evidence [56,57,68,69,70,71].

**Figure 3:**
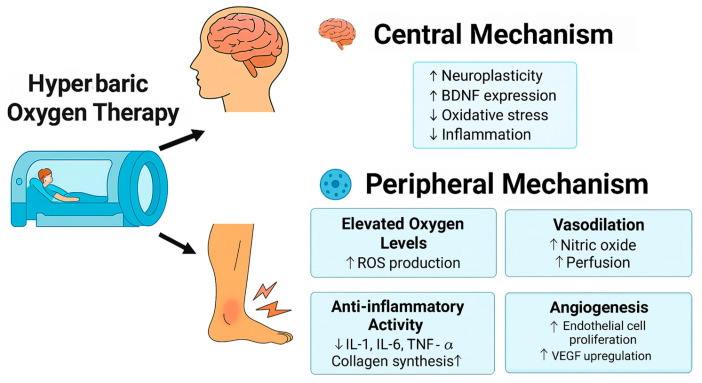
Central and peripheral mechanisms underlying the therapeutic effects of HBOT in MPS. At the central level, HBOT promotes neuroplasticity, enhances the expression of BDNF, and reduces both oxidative stress and neuroinflammation. Peripherally, HBOT increases tissue oxygenation, promotes vasodilation through nitric oxide pathways, modulates pro-inflammatory cytokines (e.g., *IL-1*, *IL-6*, *TNF-α*), stimulates angiogenesis via VEGF upregulation, and supports collagen synthesis. These synergistic mechanisms result in pain attenuation, improved functional recovery, and accelerated tissue repair.

In contrast, the evidence for HBOT in acute musculoskeletal injuries (e.g., ligament injury and DOMS) is mixed and generally less compelling. Some trials describe accelerated symptom resolution, whereas others report no meaningful difference relative to standard care [37,38,39,40,41,42,43,44]. The self-limiting nature of acute inflammation and generally limited tissue hypoxia in such injuries may reduce the clinical relevance of HBOT in these contexts, suggesting that its therapeutic window may be narrower. Consequently, no firm conclusions can currently be drawn regarding its use in acute injury management. Figure 3 provides a schematic summary of the possible mechanistic pathways, which should be regarded as provisional rather than definitive.

### 3.1. Strengths

This systematic review provides a structured synthesis of the current evidence on the use of HBOT in musculoskeletal pain-related conditions. One of the key strengths of this work lies in the comprehensive integration of findings across multiple outcome domains—including pain, fatigue, function, and recovery timelines—which allows for a holistic understanding of HBOT’s potential therapeutic role [71].

The inclusion of mechanistic insights grounded in neurophysiology and biochemistry strengthens the clinical relevance of the synthesis. Biological pathways such as oxygen-enhanced mitochondrial metabolism, the modulation of inflammatory cytokines, and the promotion of neuroplasticity offer plausible explanations for the clinical improvements reported, especially in chronic pain contexts [72,73].

Another strength is the stratified analysis by clinical scenario (e.g., chronic vs. acute *pain;* postsurgical vs. overuse *injury*), which facilitates context-specific interpretation of the findings. The detailed narrative comparison of intervention protocols (e.g., *number of sessions*, *pressure, oxygen concentration*) provides useful information for future clinical applications. Furthermore, the overall methodological quality of the included studies was low to moderate, and most studies clearly reported intervention parameters, comparators, and outcomes, which supports the transparency and reproducibility of this synthesis.

### 3.2. Limitations

Several limitations inherent to the current evidence base and to the qualitative nature of this review must be acknowledged. First, the lack of meta-analytic synthesis prevents the estimation of pooled effect sizes and limits the ability to draw statistically robust conclusions. Second, the heterogeneity of HBOT protocols represents a major source of methodological variability. Studies differed in atmospheric pressure, session frequency, total treatment duration, and timing relative to injury or diagnosis, which complicates direct comparisons and may partially account for the variability in clinical outcomes [74].

In addition, the diversity of musculoskeletal conditions—ranging from chronic pain syndromes such as Fibromyalgia syndrome to acute trauma and exercise-induced injuries—limits the external validity of the results. While chronic pain conditions consistently demonstrated benefit, findings were more mixed in postoperative recovery and largely inconclusive in acute ligamentous injuries [75].

A third limitation concerns the limited inclusion of mechanistic outcome measures. Few trials incorporated physiological, biochemical, or neuroimaging markers—such as inflammatory cytokines, electrophysiological assessments, or functional brain imaging—which restricts our ability to validate the proposed mechanisms (e.g., *central desensitization*, *neuroplasticity*, *mitochondrial enhancement*). The absence of such data hinders efforts to differentiate true biological effects of HBOT from placebo responses or natural recovery trajectories [76].

Moreover, the variability in outcome measures, follow-up durations, and comparator interventions across studies further undermines the comparability and synthesis of results. Several studies failed to report relevant co-interventions such as physical therapy, medication use, or activity restrictions, which may act as confounders influencing the observed outcomes.

Finally, the context-dependent nature of HBOT efficacy should be emphasized. For instance, the therapy appears particularly effective when applied to chronic, hypoxic, or neuroinflammatory states, yet its role in acute, self-limiting musculoskeletal conditions remains unclear. It is likely that HBOT alone may not suffice in many scenarios and that combination approaches—including structured rehabilitation programs and multimodal pain management—may be necessary to achieve optimal outcomes [77,78].

Future research should prioritize standardization of HBOT protocols, stratification by injury type and chronicity, and the integration of objective biomarkers to clarify the physiological mechanisms underpinning clinical improvement. Moreover, high-quality, multicenter RCTs with rigorous methodology and extended follow-up are essential to define the precise role of HBOT in musculoskeletal medicine and rehabilitation.

## 4. Materials and Methods

### 4.1. Data Sources and Search Strategy

This systematic review was conducted in accordance with the PRISMA (Preferred Reporting Items for Systematic Reviews and Meta-Analyses) guidelines [25]. The protocol was prospectively registered in the PROSPERO database (International Prospective Register of Systematic Reviews; registration number CRD420251073730; available at: https://www.crd.york.ac.uk/PROSPERO/view/CRD420251073730).

A comprehensive literature search was carried out between 30 June and 30 September 2025, to identify all relevant studies assessing the effectiveness of HBOT for managing MPS, with a focus on outcomes related to pain, physical performance, functionality and quality of life. The search was conducted across 5 electronic databases: MEDLINE (*PubMed*), PEDro, Scopus, CINAHL Complete, and Web of Science.

In MEDLINE (*PubMed*), the search strategy combined controlled vocabulary (MeSH terms) and free-text terms, including: “*Hyperbaric oxygenation*” [MeSH], “*Hyperbaric chamber*” [MeSH], “*Hyperbaric oxygen therapy*” [MeSH], and “*HBOT*” [tw]; these were paired with terms related to intervention and condition, such as “*Physical therapy modalities*” [MeSH], “*Conservative management*” [MeSH], “*Muscle injuries*” [MeSH], and “*Musculoskeletal diseases*” [MeSH], as well as anatomical descriptors like “*Lower extremity*”, “*Lower limb*”, and “*Leg*” [MeSH]. Equivalent strategies were adapted to meet the specific indexing and syntax requirements of each database.

Two primary and independent reviewers (A.D.R. and D.C.A.) conducted the search and selection processes. A third reviewer (C.G.C.), blinded to the selection procedure, screened titles and abstracts for eligibility and assessed full-text articles. Any discrepancies were resolved by a fourth reviewer (S.E.M.P.). Full details of the search strategies employed in each database are provided in Table S1.

### 4.2. Study Selection

Studies were considered eligible if they met the following criteria: (1) employed a randomized clinical trial, non-randomized clinical trial, or case series design; (2) were published from database inception to 1 September 2025; (3) were written in English, Spanish, or Portuguese; (4) were available in full-text format; (5) included participants diagnosed with MPS, with or without stable comorbidities, provided these conditions were reported and did not independently restrict mobility or influence pain assessment, (6) who received either HBOT or standard physiotherapy as part of conservative management or postoperative rehabilitation.

Moreover, we also included those studies which (7) reported outcomes related to muscle pain, range of motion, muscle strength, lower-limb function, or disease-specific quality of life, assessed at short-, medium-, or long-term follow-up (0–3 months, 3–6 months, or 6–12 months, respectively). When studies presented follow-up periods that overlapped two predefined intervals (e.g., 2–4 months), the study was classified according to the closest midpoint of the follow-up range. Inter-rater reliability was observed between the two primary reviewers (A.D.R. and D.C.A.) during the study, and the selection process was calculated using Cohen’s kappa coefficient, demonstrating a substantial level of agreement *κ* = 0.84 (IC 95%: 0.78–0.90).

### 4.3. Data Extraction

Data extraction was performed manually by two independent reviewers (A.D.R. and D.C.A.) using a standardized data extraction form structured around the PICO framework. No automation tools were employed at any stage of the process. The extraction form was pilot-tested on a representative subset of studies to ensure clarity, consistency, and reliability of data capture. Any discrepancies were resolved through discussion, and when necessary, a third reviewer (C.G.C.) acted as an adjudicator.

Extracted variables included: authorship, year and country of publication, study design, objectives, participant characteristics (e.g., *sample size*, *sex*, *type* and *duration of symptoms*, and *comorbidities*), details of intervention and comparator conditions, outcome measures, and main findings. To avoid unit-of-analysis errors in crossover trials, only data from the first treatment period prior to participant crossover were extracted and included in the analysis. This approach followed the methodological recommendations of the *Cochrane Handbook for Systematic Reviews of Interventions* (version 5.1.0, The Cochrane Collaboration, London, UK) [79] and prevented the double-counting of participants.

### 4.4. Methodological Quality Assessment

#### 4.4.1. Clinical Trials (PEDro Scale)

The methodological quality of the included trials was assessed using the PEDro scale [45], a validated tool for evaluating the internal validity and reporting quality of randomized controlled trials. The scale consists of 11 items, 10 of which contribute to the final score (*excluding the item related to external validity*). It examines key methodological domains such as random allocation, allocation concealment, blinding (of *participants*, *therapists*, and *assessors*), and adequacy of follow-up and data analysis.

Based on the total score (range 0–10), studies were classified as follows: 9–10 points indicated *excellent* quality, 7–8 as *moderate*, 5–6 as *good*, and ≤4 as *poor* quality. This grading facilitated a structured appraisal of the risk of bias and the overall reliability of the included evidence.

#### 4.4.2. Clinical Trials (NIH Quality Assessment Tool for Case Series Studies)

The methodological quality of case series studies was assessed using the *NIH Quality Assessment Tool for Case Series Studies* [46]. This instrument evaluates nine key domains: (1) a clearly stated study objective, (2) clear description of the study population, (3) consecutive inclusion of cases, (4) inclusion of all eligible participants, (5) clear description of the intervention or exposure, (6) consistent and reliable outcome measurement, (7) adequate follow-up period, (8) clear reporting of results, and (9) discussion of study limitations. Each criterion was rated as “*Yes*,” “*No*,” “*Partial*,” or “*Unclear*,” and an overall score was assigned to grade the methodological quality and risk of bias of the included studies. This structured approach allowed a systematic appraisal of the internal validity and reliability of the evidence from case series.

### 4.5. Risk of Bias Assessment

The risk of bias in the included randomized controlled trials was assessed using the Cochrane Risk of Bias 2.0 tool (*RoB 2.0*) [47], a standardized and validated instrument for evaluating potential sources of bias in clinical trials. This tool examines five key domains: (1) the randomization process, (2) deviations from intended interventions, (3) missing outcome data, (4) outcome measurement, and (5) selection of the reported results. Each domain was rated as presenting a “*low risk*,” “*some concerns*,” or “*high risk*” of bias, leading to an overall risk of bias judgment for each study. Any discrepancies between reviewers were solved through discussion, and when consensus could not be reached, a third reviewer (S.E.M.P.) acted as referee.

### 4.6. Certainty of Evidence: GRADE Approach

The certainty of the evidence was assessed using the Grading of Recommendations, Assessment, Development and Evaluation (GRADE) framework [48,80]. This system assesses five domains that may lead to downgrading the certainty of evidence: (1) risk of bias (e.g., lack of blinding, allocation concealment, or incomplete outcome data), (2) inconsistency (substantial heterogeneity of results across studies), (3) indirectness (differences in population, intervention, comparator, or outcomes relative to the research question), (4) imprecision (wide confidence intervals or small sample sizes), and (5) publication bias (e.g., selective reporting or absence of negative studies).

Randomized controlled trials start as high-certainty evidence, and observational studies start as low-certainty evidence. Evidence was downgraded by one level when a domain presented serious concerns and by two levels when concerns were very serious. Conversely, evidence from observational studies could be upgraded if there was (a) a large effect size; (b) evidence of a dose–response gradient; or (c) if all plausible residual confounding would reduce, rather than increase, the observed effect. Based on these considerations, the certainty of evidence for each outcome was classified into four levels: “*high*”, “*moderate*”, “*low*”, or “*very low*”, reflecting the confidence that the estimated effect is close to the true effect.

### 4.7. Data Synthesis Approach

A qualitative synthesis was performed due to the heterogeneity of study designs, intervention protocols, and outcome measures. A thematic grouping approach was applied, whereby studies were classified according to outcomes domain (pain, functional recovery, disease-specific quality of life, physiological markers and time to return-to-sport) and then by condition type (acute vs. chronic muscle injury; postoperative vs. non-surgical management). Findings within each subgroup were synthesized narratively to identify consistent patterns, divergences, and the overall direction of effects. This method aligns with established guidance for narrative synthesis in systematic reviews where meta-analysis is not feasible.

## 5. Conclusions

HBOT may provide adjunctive benefits in musculoskeletal pain syndromes, yet the current evidence remains limited. Standardized treatment protocols and high-quality trials are needed to better define its clinical applicability.

## Figures and Tables

**Table 1:** Methodological quality analysis (PEDro Scale).

Year, Author	Score	Quality	1	2	3	4	5	6	7	8	9	10	11
Zhang et al. (2025) [26]	8	Moderate	Yes	Yes	Yes	Yes	No	Yes	Yes	Yes	No	Yes	Yes
Zhu et al. (2023) [27]	6	Good	Yes	No	Yes	Yes	No	No	No	Yes	Yes	Yes	Yes
Hadanny et al. (2022) [28]	9	Excellent	Yes	Yes	Yes	Yes	Yes	Yes	Yes	Yes	No	Yes	Yes
Ince et al. (2022) [29]	6	Good	Yes	Yes	No	Yes	No	No	Yes	Yes	No	Yes	Yes
Izquierdo-Alventosa et al. (2020) [30]	9	Excellent	Yes	Yes	Yes	Yes	Yes	Yes	Yes	Yes	No	Yes	Yes
Chen et al. (2019) [31]	9	Excellent	Yes	Yes	Yes	Yes	Yes	No	Yes	Yes	Yes	Yes	Yes
Hadanny et al. (2018) [32]	7	Moderate	Yes	Yes	No	Yes	No	No	Yes	Yes	Yes	Yes	Yes
Yuan et al. (2016) [33]	3	Low	Yes	No	No	No	No	No	Yes	No	Yes	Yes	Yes
Efrati et al. (2015) [35]	6	Good	Yes	Yes	No	Yes	No	No	Yes	Yes	No	Yes	Yes
Yildiz et al. (2004) [36]	10	Excellent	Yes	Yes	Yes	Yes	Yes	Yes	Yes	Yes	Yes	Yes	Yes
Babul et al. (2003) [37]	5	Good	Yes	No	No	Yes	No	No	No	Yes	Yes	Yes	Yes
Germain et al. (2003) [38]	6	Good	Yes	Yes	No	Yes	No	No	Yes	Yes	Yes	No	Yes
Webster et al. (2002) [39]	7	Moderate	Yes	Yes	Yes	Yes	No	No	Yes	Yes	No	Yes	Yes
Harrison et al. (2001) [40]	5	Good	Yes	Yes	No	Yes	No	No	No	Yes	No	Yes	Yes
Mekjavik et al. (2000) [41]	10	Excellent	Yes	Yes	Yes	Yes	Yes	Yes	Yes	Yes	Yes	Yes	Yes
Staples et al. (1999) [42]	10	Excellent	Yes	Yes	Yes	Yes	Yes	Yes	Yes	Yes	Yes	Yes	Yes
Borromeo et al. (1997) [43]	8	Moderate	Yes	Yes	Yes	Yes	Yes	No	Yes	Yes	No	Yes	Yes
Soolsma (1996) [44]	6	Good	Yes	Yes	No	Yes	No	No	Yes	Yes	No	Yes	Yes

Methodological quality assessment of the included studies was conducted using the PEDro Scale [45]. Abbreviations: 1 = eligibility criteria (*not scored*); 2 = random allocation; 3 = concealed allocation; 4 = baseline comparability; 5 = blinding of subjects; 6 = blinding of therapists; 7 = blinding of assessors, 8 = adequate follow-up (>85%); 9 = intention-to-treat analysis; 10 = between-group statistical comparisons; 11 = reporting of point estimates and variability. The total PEDro score ranges from 0 to 10 (*excluding item 1*), with higher scores indicating greater methodological quality.

**Table 2:** NIH Quality Assessment Tool for Case Series Studies.

Year, Author	Quality	1	2	3	4	5	6	7	8	9
Botha et al. (2015) [34]	Low/moderate	Yes	Yes	Unclear	Unclear	Yes	Partial	No	Yes	Partial

Methodological quality assessment of the included studies was conducted using the NIH Quality Assessment Tool for Case Series Studies [46]. Abbreviation: 1 = Clear study objective; 2 = Study population clearly described; 3 = Consecutive inclusion of cases; 4 = Inclusion of all eligible participants; 5 = Intervention/exposure clearly described; 6 = Consistent and reliable outcome measurement; 7 = Adequate follow-up period; 8 = Results clearly reported; 9 = Study limitations discussed.

**Table 3:** Certainty of Evidence: *GRADE*.

Outcomes	Number of Studies (Subjects)	1	2	3	4	5	6	7
Pain [26,30,31,32,33,35,36,37,38,39,40,43,44]	13 (*n* = 422)	Serious *	Serious ^‡^	Not serious	Not serious	Not serious	Low	Weak in favor
Functional recovery [26,29,30,33,34,35,42,43,44]	9 (*n* = 419)	Serious *	Serious ^‡^	Not serious	Not serious	Not serious	Low	Weak in favor
Quality of life [30,31,32,35]	4 (*n* = 180)	Serious *	Serious ^‡^	Not serious	Not serious	Not serious	Low	Weak in favor
Physiological markers [26,27,28,32,37,38,40,41]	8 (*n* = 241)	Serious *	Serious ^‡^	Not serious	Not serious	Not serious	Low	Weak in favor
Return to sport [28,33,34,39,42]	5 (*n* = 159)	Serious *	Serious ^‡^	Not serious	Not serious	Not serious	Low	Weak in favor

Certainty of Evidence: *GRADE* [48]. Abbreviations: 1 = *Risk of Bias*; 2 = *Inconsistency*; 3 = *Indirectness*; 4 = *Imprecision*; 5 = *Publication Bias*; 6 = *Quality*; 7 = *Grade of Recommendation*. Note: (*) Risk of bias was rated as serious due to methodological limitations in some studies, including issues in design and implementation that may introduce bias. (^‡^) Inconsistency was rated as serious based on substantial variability across study results, possibly due to differences in populations, interventions, or measurement methods. No relevant concerns were noted for indirectness, imprecision, or publication bias. The overall quality of evidence was considered low, and the recommendations were weak in favor of the intervention.

**Table 4:** Main findings of HBOT interventions across clinical indications for MPS.

Outcomes	HBOT Protocol	Main Findings
Pain	*Fibromyalgia syndrome*: 1.45–2.4 ATA, 15–60 sessions [30,32,35,36]	↓ Pain intensity (*VAS*) ↓ Number of tender points ↑ Pain threshold
	*Exercise-induced*: 2.0–2.5 ATA, 3–40 sessions [28,31,37,38,40,42]	↓ Pain intensity (*VAS*) = No significant effect in DOMS-related conditions
	*Post-op/nerve injury*: 2.0 ATA, 5–10 sessions [26,29,44]	↓ Postoperative pain (*TKA*) ↑ Sensory recovery speed
	*Ligament injuries*: 2.0–2.5 ATA, 3–10 sessions [43,44]	= No significant effect on pain (*VAS*) ↑ Functional gains
Functional recovery	*Fibromyalgia syndrome*: 2.0 ATA, 40–60 sessions [30,32,35]	↓ Symptom severity (*FIQ*) ↑ Physical and cognitive functioning
	*Exercise/DOMS*: 2.0–2.5 ATA [28,39,42]	↑ Muscle strength recovery = No significant change in DOMS outcomes
	*Post-op/nerve repair*: 2.0 ATA [26,29,44]	↑ Range of motion (*ROM*) ↑ Mobility ↑ Muscle strength and nerve conduction (e.g., *MRC*, *ENMG*)
Quality of life	*Fibromyalgia*: 2.0 ATA [30,32,35]	↑ General quality of life (*SF-36*) ↓ Fatigue ↑ Sleep quality ↓ Psychological distress and PTSD symptoms
	*Athletes*: 2.5 ATA [28,31]	↓ Fatigue ↑ VO_2_max and anaerobic threshold (VO_2_AT) ↑ Perceived recovery
Physiological markers	2.0–2.5 ATA [26,29,32,35,41]	↓ Inflammatory biomarkers (e.g., *CRP*, *IL-6*, *TNF-α*) ↑ Nerve conduction (e.g., *ENMG*) ↑ Brain activity (e.g., *SPECT, DTI*) ↑ Tissue oxygenation (*TcPO_2_*)
Return to sport	2.0–2.5 ATA [28,29,33,39,43,44]	↑ Faster return to activity (*postoperative rehab*, *nerve repair*, *muscle injuries*) = No clear benefit in DOMS or ligament injuries

Main findings of HBOT interventions across clinical indications for musculoskeletal painful diseases. Arrows indicate the direction of observed changes: ↓ decrease; ↑ increase; = no differences. Abbreviation: DOMS: *Delayed-Onset Muscle Soreness*; DTI: *Diffusion Tensor Imaging*; ENMG: *Electroneuromyography*; FIQ: *Fibromyalgia Impact Questionnaire*; MRC: *Medical Research Council Scale*; PTSD: *Post-Traumatic Stress Disorder*; ROM: *Range of Motion*; SF-36: *Short Form Health Survey*; SPECT: *Single-Photon Emission Computed Tomography*; TcPO_2_: *Transcutaneous Oxygen Pressure*; VAS: *Visual Analog Scale*; VO_2_AT: *Anaerobic Threshold*; VO_2_max: *Maximal Oxygen Uptake*.

## Data Availability

Data supporting the reported results can be found in the manuscript.

## Supplementary Materials

The following supporting information can be downloaded at https://www.mdpi.com/article/10.3390/muscles4040063/s1. Table S1: Search Strategy, Table S2: Study Characteristics.

## Abbreviations

The following abbreviations are used in this manuscript:

HBOT	Hyperbaric Oxygen Therapy
ATA	Atmospheres Absolute
VAS	Visual Analog Scale
FIQ	Fibromyalgia Impact Questionnaire
HRQoL	Health-Related Quality of Life
EMG	Electromyography
CRP	C-Reactive Protein
IL-6	Interleukin-6
TNF-α	Tumor Necrosis Factor Alpha
SPECT	Single-Photon Emission Computed Tomography
DTI	Diffusion Tensor Imaging
DOMS	Delayed Onset Muscle Soreness
RCT	Randomized Controlled Trial
QoL	Quality of Life
MRI	Magnetic Resonance Imaging
