# Supplementary material for: Effectiveness of Hyperbaric Oxygen Therapy for Musculoskeletal Pain Syndromes: A Systematic Review
Source: Muscles. 2025 Dec 16;4(4):63. doi: 10.3390/muscles4040063 (PMC12736084; doi:10.3390/muscles4040063)
Supplement: Supplementary file 1 [file muscles-04-00063-s001.zip › muscles-3893152-supplementary.pdf]

Table S1. Search Strategy

Date	Database	Boolean Operators	Search Terms	Search Equation
14/06/2024	MEDLINE (*PubMed*)	AND, OR	"Musculoskeletal Diseases"[Mesh]; "Muscles/injuries"[Mesh]; "Lower Extremity"[Mesh]; "Physical Therapy Modalities"[Mesh]; "Hyperbaric Oxygenation"[Mesh]	("Physical Therapy Modalities"[Mesh] OR "Hyperbaric Oxygenation"[Mesh]) AND ("Muscles/injuries"[Mesh] OR "Musculoskeletal Diseases"[Mesh]) AND "Lower Extremity"[Mesh]
25/06/2024	MEDLINE (*PubMed*)	AND, OR	"Hyperbaric oxygenation"[Tiab]; "Hyperbaric chamber"[Tiab]; "Muscle injury"[Mesh]; "Pain"[Mesh]; "Lower limb"[Mesh]	("Hyperbaric oxygenation"[Tiab] OR "Hyperbaric chamber"[Tiab]) AND "Muscle injury"[Mesh] AND "Pain"[Mesh] AND "Lower limb"[Mesh]
02/07/2024	MEDLINE (*PubMed*)	AND, OR	"Hyperbaric Oxygenation"[Mesh]; "Hyperbaric Chambers"[Mesh]; "Muscle Injuries"[Mesh]; "Lower Extremity"[Mesh]; "Pain Management"[Mesh]; "Rehabilitation"[Mesh]	"Hyperbaric Oxygenation"[Mesh] OR "Hyperbaric Chambers"[Mesh]) AND ("Muscle Injuries"[Mesh] AND "Lower Extremity"[Mesh]) AND ("Pain Management"[Mesh] OR "Rehabilitation"[Mesh])
14/06/2024	PEDro	AND, OR	"hyperbaric oxygen"[Mesh]; "hyperbaric chamber"[Mesh]; "hyperbaric oxygen therapy"[Tiab]; "muscle injury"[Mesh]; "musculoskeletal diseases"[Mesh]; "lower extremity"[Mesh]; "lower limb"[Tiab]; "physical therapy"[Mesh]	("hyperbaric oxygen" OR "hyperbaric chamber" OR "hyperbaric oxygen therapy") AND ("muscle injury" OR "musculoskeletal diseases") AND ("lower extremity" OR "lower limb") AND "physical therapy"
25/06/2024	PEDro	AND	"hyperbaric oxygen"[Mesh]; "rehabilitation"[Mesh]; "muscle injury"[Mesh]; "pain"[Mesh]	"hyperbaric oxygen" AND "rehabilitation" AND "muscle injury" AND "pain"
02/07/2024	PEDro	AND, OR	"hyperbaric oxygen therapy"[Tiab]; "hyperbaric chamber"[Mesh]; "lower limb"[Mesh]; "sports injuries[Mesh]"	("hyperbaric oxygen therapy" OR "hyperbaric" chamber") AND "lower limb" AND "sports injuries"
14/06/2024	Scopus	AND, OR	TITLE-ABS-KEY("hyperbaric oxygen"); TITLE-ABS-KEY("hyperbaric chamber"); TITLE-ABS-KEY("hyperbaric oxygen therapy"); TITLE-ABS-KEY("muscle injury"); TITLE-ABS-KEY("musculoskeletal diseases"); TITLE-ABS-	TITLE-ABS-KEY(("hyperbaric oxygen" OR "hyperbaric chamber" OR "hyperbaric oxygen therapy") AND ("muscle injury" OR "musculoskeletal diseases") AND ("lower extremity" OR "lower limb"))

			KEY("lower extremity"); TITLE-ABS-KEY("lower limb")	
25/06/2024	Scopus	AND	TITLE-ABS-KEY("hyperbaric oxygen therapy"); TITLE-ABS-KEY("pain management"); TITLE-ABS-KEY("rehabilitation")	TITLE-ABS-KEY("hyperbaric oxygen therapy" AND "pain management" AND "rehabilitation")
02/07/2024	Scopus	AND, OR	TITLE-ABS-KEY("hyperbaric chamber"); TITLE-ABS-KEY("hyperbaric oxygen"); TITLE-ABS-KEY("sports injuries"); TITLE-ABS-KEY("lower limb")	TITLE-ABS-KEY(("hyperbaric chamber" OR "hyperbaric oxygen") AND "sports injuries" AND "lower limb")
14/06/2024	CINAHL Complete	AND, OR	MH "Hyperbaric Oxygenation"; "hyperbaric chamber"; MH "Muscle Injuries"; "musculoskeletal diseases"; MH "Lower Extremity"; "lower limb"	(MH "Hyperbaric Oxygenation" OR "hyperbaric chamber") AND (MH "Muscle Injuries" OR "musculoskeletal diseases") AND (MH "Lower Extremity" OR "lower limb")
25/06/2024	CINAHL Complete	AND	MH "Hyperbaric Oxygenation"; MH "Rehabilitation"; MH "Pain"	(MH "Hyperbaric Oxygenation") AND (MH "Rehabilitation") AND (MH "Pain")
02/07/2024	CINAHL Complete	AND, OR	MH "Hyperbaric Oxygenation"; "hyperbaric chamber"; MH "Sports Injuries"; MH "Lower Extremity"	(MH "Hyperbaric Oxygenation" OR "hyperbaric chamber") AND (MH "Sports Injuries") AND (MH "Lower Extremity")
14/06/2024	Web of Science Core Collection	AND, OR	TS=("hyperbaric oxygen"); TS=("hyperbaric chamber"); TS=("muscle injury"); TS=("musculoskeletal diseases"); TS=("lower extremity"); TS=("lower limb")	TS=(("hyperbaric oxygen" OR "hyperbaric chamber") AND ("muscle injury" OR "musculoskeletal diseases") AND ("lower extremity" OR "lower limb"))
25/06/2024	Web of Science Core Collection	AND	TS=("hyperbaric oxygen therapy"); TS=("pain management"); TS=("rehabilitation")	TS=("hyperbaric oxygen therapy" AND "pain management" AND "rehabilitation")
02/07/2024	Web of Science Core Collection	AND, OR	TS=("hyperbaric chamber"); TS=("hyperbaric oxygen"); TS=("sports injuries"); TS=("lower limb")	TS=(("hyperbaric chamber" OR "hyperbaric oxygen") AND "sports injuries" AND "lower limb")

Note: The search strategies were conducted between June 14 and August 10, 2024, across several biomedical databases. Boolean operators ("AND", "OR") were used, along with MeSH descriptors and free-text terms (Tiab) related to muscle injuries, lower limb conditions, physical therapy modalities, and hyperbaric oxygen therapy. Search equations were tailored to the syntax and indexing system of each database.

Table S2. Study Characteristics

Author (Year)	Study Design	Participants	Intervention	Comparison	Outcomes	Conclusion
Zhang et al. (2025) [32]<br><br>China	RCT, single-center, patient- and assessor-blinded	N=80 undergoing **TKA for osteoarthritis** (HBOT = 40, CG=40)	HBOT post-op group: (2.0 ATA, 100% O_2, 5 sessions)	Control group: Normobaric oxygen	**Muscle Damage Markers (Day 3 post-op)**<br>• CK:<br>○ HBOT=336.5 ± 106.2 U/L vs CG=483.3 ± 128.1; p<0.001<br>• LDH:<br>○ HBOT=203.7 ± 56.4 vs CG=273.5 ± 70.2; p<0.001<br>• Myoglobin:<br>○ HBOT=214.8 ± 78.5 vs CG=324.6 ± 92.3; p<0.001<br>• GOT:<br>○ HBOT=19.4 ± 6.2 vs CG=26.7 ± 9.1; p<0.001<br>**Inflammatory Markers (Day 3 post-op)**<br>• CRP:<br>○ HBOT=43.2 ± 10.5 mg/L vs CG=58.9 ± 12.6; p<0.001<br>• IL-6:<br>○ HBOT=37.6 ± 9.8 pg/mL vs CG=50.1 ± 11.3; p<0.001<br>• TNF-α:<br>○ HBOT=12.4 ± 3.7 pg/mL vs CG=16.1 ± 4.5; p=0.002<br>**Quadriceps Strength (Recovery % baseline)**<br>• POD 14:<br>○ HBOT=82.3 ± 9.5% vs CG=72.6 ± 11.1%; p=0.001<br>**Swelling (Thigh Circumference Ratio)**<br>• POD 3:<br>○ HBOT=1.05 ± 0.03 vs CG=1.11 ± 0.04; p<0.001<br>**VAS Pain (Rest/Movement)**<br>• Day 2-3:<br>○ HBOT significantly lower (exact values not reported); p<0.05.	HBOT after TKA reduces muscle damage and inflammation, improves strength recovery and swelling, and relieves pain in the early postoperative phase.

Author (Year)	Study Design	Participants	Intervention	Comparison	Outcomes	Conclusion
Zhu et al. (2023) [33]<br><br>China	RCT, single-blind	N=18 skeleton athletes with **overuse injuries** (HBOT = 8, CG=8)	HBOT group: (1.3 ATA, 60 min, 4×/week, 4 weeks) + physical training)	Control group: Physical training only	**Oxidative Stress**<br>• MDA:<br>○ HBOT=5.82 (0.99) vs CG=8.84 (1.07); $p<0.05$<br>○ PC: EG=1.06 (0.08) vs CG=1.27 (0.09); $p<0.05$<br>**Antioxidant Activity**<br>• SOD:<br>○ HBOT=19.96 (1.42) vs CG=15.96 (1.82); $p<0.05$<br>• CAT:<br>○ HBOT=8.34 (0.46) vs CG=5.98 (0.84); $p<0.05$<br>• T-AOC:<br>○ HBOT=0.90 (0.07) vs CG=0.66 (0.07); $p<0.05$<br>**Fatigue Marker**<br>• CK:<br>○ HBOT =192.19 (27.39) vs CG=280.50 (41.05); $p<0.05$	HBOT significantly reduced oxidative stress, increased antioxidant enzymes, and lowered exercise-induced fatigue.
Hadanny et al. (2022) [34]<br><br>Israel	Double-blind, randomized, placebo-controlled trial	N = 37 **athletes with DOMS aferte exercise** aged 40–50 years, training ≥4 times/week	HBOT group: 40 sessions of 100% O_2 at 2 ATA (60 min) 8 weeks	Sham group: 40 sessions of air at 1.02 ATA (60 min) 8 weeks	**VO_2max (mL/min)**<br>• HBOT: 2956.19 (540.85) vs CG (SHAM): 2631.07 (751.31); p = 0.010<br>**VO_2 en el umbral anaeróbico – VO_2AT (mL/min)**<br>• HBOT: 1326.56 (249.11) vs CG (SHAM): 1208.53 (266.18); p = 0.026<br>**Potencia máxima (Watts)**<br>• HBOT: 290.93 (51.87) vs CG (Sham group): 254.73 (68.25); p = 0.030	HBOT improves aerobic fitness (VO_2max, VO_2AT, power) in master athletes. Benefits are linked to increased mitochondrial respiration and mass.

Author (Year)	Study Design	Participants	Intervention	Comparison	Outcomes	Conclusion
Ince et al. (2022) [35]<br><br>Turkey	Prospective comparative study	N = 74 patients with **Ulnar and Median nerve injuries** (38 HBOT vs 36 control)	HBOT group: 5 daily sessions of 2 ATA, 2 h/day, starting on day 1 after epineural repair<br><br>Follow-up: 3, 6, 12 months; 2 and 3 years for elbow-level injuries	Control group: Epineural repair only	**ENMG – 3 months (distal forearm)**<br>**Ulnar nerve (conduction velocity, m/s)**<br>● HBOT: 48.8 vs CG: 15.1; p = 0.011<br>**Median nerve (conduction velocity, m/s)**<br>● HBOT: 43.4 vs CG: 29.0; p = 0.030<br>**Median nerve (latency, ms)**<br>● HBOT: 9.5 vs CG: 19.1; p = 0.041<br>**ENMG – 6 months (latency, ms)**<br>● Ulnar nerve<br>○ HBOT: 3.58 vs CG: 5.70; p = 0.028<br>● Median nerve<br>○ HBOT: 3.20 vs CG: 5.20; p = 0.022<br>**ENMG – 2 years (elbow, ulnar nerve)**<br>● Conduction velocity (m/s)<br>○ HBOT: 54.3 vs CG: 38.2; p = 0.042<br>**Two-point discrimination (12 months, fingertip, mm)**<br>● HBOT: 4.1 (2.9) vs CG: 5.4 (2.7); p < 0.05<br>**Muscle strength (MRC scale, 12 months, elbow – ulnar nerve)**<br>● HBOT: M4 vs CG: M2; p < 0.05 (qualitative)	Early HBOT (5 daily sessions starting on day 1) after primary repair of median and ulnar nerve injuries
Izquierdo-Alventosa et al. (2020) [36]<br><br>Spain	RCT, double-blind	N = 49 women with **fibromyalgia syndrome** (HBOT = 17, Exercise=16, CG=16)	HBOT group: (1.45 ATA, 90 min, 5×/week, 40 sessions) or resistance training)	Control group: No intervention	**Pain (VAS)**<br>● HBOT: 4.88 (2.32) vs CG: 5.5 (2.25); Exercise: 5.38 (2.16)<br>**Pressure Pain Threshold (PPT)**<br>● Lateral epicondyle:<br>○ HBOT: 1.75 (0.48) vs CG: 1.24 (0.48)<br>● Gluteal:<br>○ Exercise: 2.72 (2.14) vs CG: 1.68 (0.73)<br>**Fatigue (6MWT)**<br>● Induced fatigue score:<br>○ HBOT: 6.76 (2.14) vs CG: 6.75 (2.62)<br>**Distance walked**<br>○ HBOT: 558.29 (68.83) vs CG: 497.31 (76.29)<br>**Physical Performance (SPPB)**<br>○ HBOT: 0.89 (0.23) vs Exercise: 1.04 (0.23)	HBOT and exercise improved PPT, endurance and function. Only HBOT improved perceived fatigue and pain at rest.

Author (Year)	Study Design	Participants	Intervention	Comparison	Outcomes	Conclusion
Chen et al. (2019) [37]<br><br>China	RCT, double-blind	N = 41 elite athletes with **exercise-induced pain** (HBOT = 20, CG=21)	HBOT group: (2.5 ATA) + pure air	Placebo HBOT group: (1.3 ATA, ambient air)	**Pain Reduction**<br>● VAS:<br>○ HBOT=1.4 (1.7) vs CG=3.3 (1.8); $p<0.05$<br>**Interference with Activities**<br>● General:<br>○ HBOT=0.8 (1.6) vs CG=2.1 (2.4)<br>● Sleep:<br>○ HBOT=1.3 (1.6) vs CG=3.2 (1.4)<br>● Quality of life:<br>○ HBOT=1.1 (1.6) vs CG=3.0 (1.5)	HBOT significantly reduced pain interference and improved sleep and QoL in elite athletes.
Hadanny et al. (2018) [38]<br><br>Israel	Prospective, randomized controlled crossover trial	N = 30 women diagnosed with **Fibromyalgia syndrome** according to ACR criteria	HBOT group: (60 daily sessions, 100% oxygen, 90 min/session, air breaks every 20 min; pressure ~2.0 ATA (not specified).	Control group: Psychotherapy during the first phase, then crossed over to receive HBOT	**Widespread pain (WPI)**:<br>● HBOT = 15.27 ± 1.6 vs CG = 7.53 ± 3.7 ($p < 0.001$)<br>**Symptom severity (SSS):**<br>● HBOT = 11.5 ± 1.8 vs CG = 5.13 ± 2.8 ($p < 0.001$)<br>**Functional impact of fibromyalgia (FIQ):**<br>● HBOT = 75.6 ± 15.5 vs CG = 48.2 ± 21.3 ($p < 0.001$)<br>**Perceived physical health (SF-36 Physical):**<br>● HBOT = 37.2 ± 8.3 vs CG = 46.5 ± 9.1 ($p < 0.001$)<br>**Perceived mental health (SF-36 Mental):**<br>● HBOT = 31.8 ± 9.2 VS CG = 41.3 ± 8.9 ($p < 0.001$)<br>**Post-traumatic stress symptoms (CAPS):**<br>● HBOT 68.2 ± 16.3 vs CG = 39.3 ± 18.4 ($p < 0.001$)<br>**Global psychological distress (SCL-90 GSI):**<br>● HBOT = 1.97 ± 0.6 vs CG = 1.23 ± 0.6 ($p < 0.001$)<br>**Regional brain activity (SPECT):**<br>● HBOT ↑ activity in BA 9, 10, 11, 25; ↓ in BA 7, 31, 36 ($p < 0.05$).<br>**White matter integrity DTI (FA):**<br>● Anterior thalamic radiation: 0.46 → 0.51 ($p = 0.0001$)<br>● Left insula: 0.42 → 0.45 ($p = 0.001$)<br>● Right thalamus: 0.49 → 0.53 ($p = 0.001$)	HBOT improved pain, function, and psychological symptoms in women with fibromyalgia and childhood trauma, alongside neuroplastic changes on SPECT and DTI.

Author (Year)	Study Design	Participants	Intervention	Comparison	Outcomes	Conclusion
Yuan et al. (2016) [39]<br><br>China	RCT (case comparison)	N=2 with **grade I ankle sprain** (HBOT =1, CG=1)	HBOT group: (2.5 ATA, 60 min, 5 days) + rehab for ankle sprain	Control group: Rehab only	**Isokinetic Strength (Peak Torque, Nm)**<br>● Dorsiflexion:<br>○ HBOT = 27.3 vs CG = 16.2<br>● Plantarflexion:<br>○ HBOT = 16.1 vs CG = 11.4<br>● Eversion:<br>○ HBOT = 11.6 vs Control = 8.0<br>● Inversion:<br>○ HBOT = 10.7 vs Control = 8.2<br>**Time to Peak Torque (ms)**<br>● Dorsiflexion:<br>○ HBOT = 75.0 vs Control = 225.0<br>● Plantarflexion:<br>○ HBOT = 220.0 vs Control = 185.0<br>● Eversion:<br>○ HBOT = 190.0 vs Control = 245.0<br>● Inversion:<br>○ HBOT = 230.0 vs Control = 300.0<br>**Average Power (W)**<br>● Dorsiflexion:<br>○ HBOT = 25.1 vs Control = 10.8<br>● Plantarflexion:<br>○ HBOT = 10.6 vs Control = 5.4<br>● Eversion:<br>○ HBOT = 9.9 vs Control = 6.1<br>● Inversion:<br>○ HBOT = 7.4 vs Control = 6.2<br>**Pain (VAS):**<br>● Lower in the HBOT patient from Day 2 onwards	HBOT + rehab more effective in reducing pain and improving strength vs rehab alone.
Botha et al. (2015) [40]<br><br>South Africa	Retrospective case series	N = 42 rugby players with **hamstring injuries** (37 grade I, 5 grade II)	HBOT + PRP group: (2.2 ATA, 11 sessions)	Control group: PRP + rehab	**Recovery Time**<br>**Grade I hamstring injuries:**<br>● HBOT + PRP = 13.1 ± 6.4 days vs Standard (CG) = 21 days ($p < 0.05$)<br>**Grade II hamstring injuries:**<br>● HBOT + PRP = 22.8 ± 8.7 days vs Standard (CG) = 42 days ($p < 0.05$)	HBOT + PRP reduced healing time and recurrence in hamstring injuries.

Author (Year)	Study Design	Participants	Intervention	Comparison	Outcomes	Conclusion
Efrati et al. (2015) [41]<br><br>Israel	Prospective, active control, crossover clinical trial	N = 60 women with **Fibromyalgia Syndrome**, aged 21–67 years. Final analysis: N = 48 (HBOT = 24; Crossover Group = 24)	HBOT group: (40 sessions, 5 days/week, 90 minutes per session, 100% oxygen at 2.0 ATA)	Crossover group: evaluated after a 2-month control period without treatment before receiving HBOT Treated group: evaluated pre- and post-HBOT	**Tender Point Count**<br>• HBOT = 17.33 → 8.87; $p < 0.001$<br>• CG = 17.71 → 17.24, $p < 0.001$<br>**Dolorimeter Threshold (kg/cm²)**<br>• HBOT =0.55 → 1.65; $p < 0.001$<br>• CG = 0.72 → 0.58, $p < 0.001$<br>**Functional Impairment (FIQ Score)**<br>• HBOT = 3.76 → 2.51; $p < 0.001$<br>• CG = 3.76 → 3.7, $p = 0.05$<br>**Psychological Distress (SCL-90 Score)**<br>• HBOT =0.88 → 0.66; $p = 0.004$<br>• CG = 1.23 → 1.08; $p < 0.01$<br>**Quality of Life (SF-36 Score)**<br>• HBOT = 3.15 → 3.48; $p < 0.001$<br>• CG = 2.89 → 3.03; $p < 0.001$<br>**Brain Activity (SPECT Imaging)**<br>After HBOT:<br>• ↑ Increased activity in frontal Brodmann areas: BA 9, 10, 11, 25<br>• ↓ Decreased activity in posterior areas: BA 7, 17, 36, 37	HBOT significantly improved pain, function, psychological distress, and quality of life in women with FMS. It also induced neuroplastic changes, normalizing brain activity in pain-related regions.
Yildiz et al. (2004)[42]<br><br>Turkey	RCT, double-blind	N=50 patients with **Fibromyalgia syndrome** (HBOT = 26, CG=24)	HBOT group: (2.4 ATA, 90 min, 15 sessions)	HBOT placebo group: (1 ATA)	**Tender Points**<br>• HBOT =6.04 (1.18) vs CG=12.54 (1.10); $p<0.05$<br>**Pain Threshold (algometer)**<br>• HBOT =1.33 (0.12) vs CG=0.84 (0.12); $p<0.05$<br>**Pain (VAS)**<br>• HBOT = 31.54 (8.34) vs CG=55.42 (6.58); $p<0.05$	HBOT significantly improved fibromyalgia symptoms.

Babul et al. (2003) [43]<br><br>Canada	RCT, double-blind	N=16 with **DOMS after eccentric exercise** (HBOT = 8, CG=8)	HBOT + Eccentric contractions group:<br>(2 ATA, 60 min, 4 days)	Same protocol + placebo HBOT	**Pain (VAS)**<br>● Day 1 (baseline): HBOT = 0.00 ± 0.00 vs CG = 0.00 ± 0.00 ($p > 0.05$)<br>● 4 h post-exercise (Day 2): HBOT = 0.15 ± 0.87 vs CG = 0.00 ($p > 0.05$)<br>● 24 h post (Day 3): HBOT = −0.65 ± 0.73 vs CG = 0.00 ($p > 0.05$)<br>● 48 h post (Day 4): HBOT = 1.75 ± 0.79 vs CG = 0.00 ($p > 0.05$*)<br>● 72 h post (Day 5): HBOT = 1.35 ± 0.59 vs CG = 0.00 ($p > 0.05$*)<br>**Eccentric strength**<br>● Day 1: HBOT = −6.38 ± 15.64 vs CG = 0 ($p > 0.05$)<br>● Day 2 (4 h post): HBOT = −18.50 ± 12.02 vs CG = 0 ($p > 0.05$)<br>● Day 3: HBOT = −25.75 ± 12.64 vs CG = 0 ($p > 0.05$)<br>● Day 4: HBOT = −30.00 ± 13.93 vs CG = 0 ($p > 0.05$*)<br>● Day 5: HBOT = −28.25 ± 13.97 vs CG = 0 ($p > 0.05$)<br>**CK & MDA levels**<br>● Serum creatine kinase (U/L)<br>○ Day 1: HBOT = −246.0 ± 323.9 vs CG = 0 ($p > 0.05$)<br>○ Day 2: HBOT = −68.3 ± 166.8 vs CG = 0 ($p > 0.05$)<br>○ Day 5: HBOT = 43.6 ± 61.2 vs CG = 0 ($p > 0.05$)<br>● Malondialdehyde (nmol/mL)<br>● Day 1: HBOT = −0.29 ± 0.61 vs CG = 0 ($p > 0.05$)<br>● Day 2: HBOT = −0.55 ± 0.58 vs CG = 0 ($p > 0.05$)<br>● Day 5: HBOT = −0.44 ± 0.57 vs CG = 0 ($p > 0.05$)<br>**Quadriceps circumference (cm)**<br>● At 10 cm above the patella<br>○ Day 1: HBOT = −0.43 ± 2.81 vs CG = 0 ($p > 0.05$)<br>○ Day 5: HBOT = −0.66 ± 2.73 vs CG = 0 ($p > 0.05$)	HBOT was not effective for post-exercise muscle damage recovery.

Author (Year)	Study Design	Participants	Intervention	Comparison	Outcomes	Conclusion
					• At 20 cm above the patella<br>○ Day 1: HBOT = −0.99 ± 2.93 vs CG = 0 ($p > 0.05$)<br>○ Day 5: HBOT = −1.02 ± 2.94 vs CG = 0 ($p > 0.05$)<br>**MRI inflammation markers**<br>• MRI T2 relaxation times<br>○ **Rectus femoris (Day 5):**<br>■ HBOT = −0.06 ± 0.21 vs CG = 0 ($p > 0.05$)<br>○ **Vastus intermedius (Day 5):**<br>■ HBOT = 0.26 ± 0.16 vs CG = 0 ($p > 0.05$)<br>○ **Vastus lateralis (Day 5):**<br>■ HBOT = −0.04 ± 0.11 vs CG = 0 ($p > 0.059$)	
Germain et al. (2003) [44]<br><br>Canada	RCT	N=16 with **quadriceps DOMS** (EG=8, CG=8)	HBOTgroup: (2.0 ATA, 100% O_2, 5 daily sessions post-exercise)	Sham group: air sessions	**Muscle soreness (VAS):**<br>• No significant differences ($p > 0.05$)<br>**Isometric strength (Nm): No significant difference**<br>• No significant differences ($p > 0.05$)<br>**Muscle circumference (cm)**<br>• No significant differences ($p > 0.05$)<br>**MRI signal intensity (T2):**<br>• No significant differences ($p > 0.05$)<br>**Oxidative stress markers:**<br>• No significant differences ($p > 0.05$)	HBOT did not provide any significant benefit in the treatment of delayed-onset muscle soreness (DOMS).
Webster et al. (2002) [45]<br><br>Canada	RCT, single-blind	N=12 with **gastrocnemius strain** (EG=6, CG=6)	HBOT (2.5 ATA, 100% O_2, 3 sessions post-exercise)	Sham air treatment (1.3 ATA)	**Isometric peak torque (Nm):**<br>• HBOT = 81.5 (10.1) vs CG=67.2 (13.3); $p<0.05$<br>**Pain VAS (0–10):**<br>• HBOT = 3.1 (1.1) vs CG=4.8 (1.2); $p<0.05$<br>**CSA via MRI (cm^2):**<br>• No significant differences ($p > 0.05$)<br>**Isokinetic torque, endurance:**<br>• No significant differences ($p > 0.05$)	HBOT significantly improved isometric strength and reduced perceived pain, but had no effect on muscle cross-sectional area or other strength metrics.

Author (Year)	Study Design	Participants	Intervention	Comparison	Outcomes	Conclusion
Harrison et al. (2001) [46]<br><br>USA	Randomized controlled trial (3 groups)	N=20 with **exercise-induced muscle injury** (HBOT immediate=6, HBOT delayed=7, CG=7)	HBOT group: 100 min daily, 2.5 ATA, 100% oxygen with intermittent room air, for 5 days	No treatment	**Isometric strength (Day 15, % of baseline):**<br>● iHBO: 88.2 (18.7) vs dHBO: 79.2 (34.4) vs CG: 80.2 (23.5); p = 0.459<br>**CSA (Day 2, mm²):**<br>● iHBO: 2329.2 (476.9) vs dHBO: 2563.7 (645.3) vs CG: 2239.7 (315.8); p = 0.438<br>**T2 relaxation (Day 7, ms):**<br>● iHBO: 47.5 (15.4) vs dHBO: 44.1 (10.5) vs CG: 38.7 (8.1); p = 0.692<br>**Serum CK and soreness**:<br>● Significant changes over time but no group differences.	HBOT, whether applied immediately (2 h) or delayed (24 h), did not improve recovery from exercise-induced muscle injury.
Mekjavic et al. (2000) [47]<br><br>Slovenia	RCT, single-blind	N=24 men with **eccentric arm injury** (HBOT = 12, CG=12)	Eccentric training + HBOT (2.5 ATA, 60 min, 6 days)	Same protocol + hypoxic placebo gas	**TcPO2**<br>• HBOT =1420 ± 144 mmHg vs CG=91 ± 23 mmHg<br>**Muscle Strength (MVC)**<br>• HBOT = drop: 5.1 ± 3.8 kp vs CG drop = 24.6 ± 3.4 kp<br>**VAS Pain / Arm Circumference**<br>• No significant differences	HBOT improved oxygenation but not pain or muscle recovery.
Staples et al. (1999) [48]<br><br>Australia	RCT, double-blind	N=66 with **quadriceps DOMS** (Phase1=36, Phase2=30)	HBOT post-exercise (2 ATA)	Simulated treatment (1.2 ATA)	**Torque Recovery**<br>• F1: HBOT=69.2 Nm vs Sim=49.6 Nm; *p*=0.021<br>• F2: HBOT-5d=191.9 Nm vs Sim=156.6 Nm; *p*=0.005<br>**VAS Pain**<br>• No significant differences ($p > 0.05$)	HBOT improves eccentric torque recovery post-exercise; no effect on perceived pain.
Borromeo et al. (1997) [49]<br><br>USA	RCT	N=32 with **acute ankle sprain** (EG=16, CG=16)	HBOT (2.0 ATA, 100% O_2, 3 sessions within 72 hours)	Sham air sessions (1.1 ATA)	**Functional index (day 7):**<br>● HBOT =83.7 (11.6) vs CG=73.9 (14.6); p=0.037<br>**Pain (VAS):**<br>● HBOT =1.7 (1.7) vs CG=2.1 (1.5); $p > 0.05$<br>**Ankle circumference (cm):**<br>● HBOT =23.4 (2.1) vs CG=23.5 (1.9); $p > 0.05$<br>**Dorsiflexion ROM (°):**<br>● HBOT =12.2 (4.1) vs CG=12.1 (4.2); $p > 0.05$<br>**Pain-free walking time (days):**<br>● HBOT =7.6 (2.4) vs CG=8.3 (2.8); $p > 0.05$	HBOT significantly improved functional outcomes but had no statistically significant effect on pain, swelling, ROM, or recovery time.

Author (Year)	Study Design	Participants	Intervention	Comparison	Outcomes	Conclusion
Soolsma (1996) [50]<br><br>Canada	RCT	N=14 patients with **Grade II MCL injury** (HBOT =7, CG=7)	HBOT (2.5 ATA, 100% O_2, 10 sessions	No HBOT	**Edema (cm^3):**<br>• HBOT = 31.57 (9.56) vs CG=38.43 (9.76); p=0.045<br>**Muscle wasting (cm):**<br>• HBOT = 0.50 (0.56) vs CG=1.36 (0.59); p=0.023<br>**Max knee flexion (°):**<br>• HBOT = 125.71 (13.65) vs CG=111.14 (8.72); p=0.039<br>**Knee ROM (°):**<br>• HBOT = 38.00 (12.86) vs CG=25.86 (8.29); p=0.041<br>**Pain (VAS):**<br>• HBOT = 1.71 (1.38) vs CG=1.86 (1.46); p=0.829<br>**One-legged hop test (cm):**<br>• HBOT = 67.71 (6.52) vs CG=68.57 (6.79); p=0.797	HBOT significantly reduced edema and muscle atrophy, and improved ROM and knee flexion, but had no significant effect on pain or hopping performance.

Table S2. Study Characteristics. **Abbreviation: ATA** (atmospheres absolute), **ACR** (American College of Rheumatology), **BA** (Brodmann area), **CAPS** (Clinician-Administered PTSD Scale), **CAT** (catalase), **CK** (creatine kinase), **CRP** (C-reactive protein), **CSA** (cross-sectional area), **DTI** (diffusion tensor imaging), **DOMS** (delayed-onset muscle soreness), **EG** (experimental group), **FIQ** (Fibromyalgia Impact Questionnaire), **GOT** (glutamate oxaloacetate transaminase), **GSI** (Global Severity Index), **HBOT** (hyperbaric oxygen therapy), **IL-6** (interleukin-6), **LDH** (lactate dehydrogenase), **MDA** (malondialdehyde), **MRI** (magnetic resonance imaging), **Nm** (newton-metre), **NS** (not significant), **PC** (protein carbonyls), **POD** (postoperative day), **PRP** (platelet-rich plasma), **QoL** (quality of life), **RCT** (randomized controlled trial), **ROM** (range of motion), **SCL-90** (Symptom Checklist-90), **SF-36** (Short Form 36 Health Survey), **SOD** (superoxide dismutase), **SPPB** (Short Physical Performance Battery), **SSS** (Symptom Severity Scale), **T-AOC** (total antioxidant capacity), **$TcPO_2$** (transcutaneous partial pressure of oxygen), **TKA** (total knee arthroplasty), **TNF-α** (tumor necrosis factor-alpha), **VAS** (visual analog scale) and **WPI** (Widespread Pain Index).
